# Standards of Care for Children in Emergency Departments: International Federation of Emergency Medicine Agenda for the Care of Children

**DOI:** 10.5811/westjem.2020.2.46917

**Published:** 2020-04-21

**Authors:** Camilo E. Gutierrez, Marianne Gausche-Hill, Rodrick K. Lim

**Affiliations:** * Children’s National Hospital, George Washington University School of Medicine and Health Sciences, Division of Emergency Medicine and Trauma Center, Washington, District of Columbia; † Los Angeles County Emergency Medical Services Agency, Harbor-UCLA Medical Center, David Geffen School of Medicine at UCLA, Department of Emergency Medicine, Los Angeles, California; ‡ Children’s Hospital at LHSC, Department of Pediatrics and Medicine, Schulich School of Medicine & Dentistry-Western University, London, Ontario, Canada

Pediatric emergency medicine (PEM) is a relatively recent subspecialty, recognized in the United States in 1992.^1^ This came almost 13 years after emergency medicine (EM) was recognized as a specialty,^2^ and after the healthcare system recognized that the care of children requires specific knowledge and skills different from adults. Over the next decade, PEM progressed in the US and in parallel in Canada, the United Kingdom, and Australia as EM systems matured. To date, PEM is still an under-represented specialty, with only a dozen countries recognizing it as a distinct specialty, and only half of those offering accredited PEM training program.

Regardless of the maturity of PEM systems in developing countries, there is widespread disparity in mortality by geography and income. The burden of deaths of children in regions of the world such as sub-Saharan Africa and South Asia^3^ is overwhelming. It is estimated that, in a given year, almost 4.5 million children under five would have survived had the mortality rate in their country been as low as the lowest in their region, and lower still if they could match that of Australia and New Zealand.^4^

The International Federation of Emergency Medicine (IFEM) is the umbrella association of EM globally, and is composed of over 60 national EM associations.^5^ IFEM represents a coordinated consortium of these organizations with the vision to lead, promote access, and advance the growth of EM. The Pediatric Emergency Medicine Special Interest Group (PEMSIG) is one of the leading special-interest groups in IFEM. Part of its mission is to promote best practices in PEM education and training, as well as aid in the development of PEM globally. PEMSIG achieves this through promoting the need for specialized care for children, and supporting individuals and societies to develop acute care systems for the care of children.

As part of its effort to support and improve pediatric care globally, PEMSIG developed the third revision of the *Standards for the Care of Children in the Emergency Department* (*StandardsV3*).^6^ In this document, PEMSIG offers a thorough examination of the key aspects of emergency care of children and offers recommendations regarding the standards that should be attained by those managing emergency departments where children are seen. Recommendations include that emergency clinicians be aware of issues around consent for care, reporting of child maltreatment, and safe discharge of children. They also address the need for education of emergency clinicians in the immediate care of children requiring resuscitation, and establishing information systems, data collection, and quality improvement processes. A complete list of essential and aspirational recommendations is found in the *StandardsV3* document (https://www.ifem.cc/wp-content/uploads/2019/06/Standards-of-Care-for-Children-in-Emergency-Departments-V3-2019.pdf).^6^

It is important to understand that these *StandardsV3* are not the ultimate and most comprehensive guideline for pediatric emergency care. Rather, they form the foundation by providing recommendations and standards for any clinician and service that cares for children. Our hope is that the promulgation and dissemination of these *StandardsV3* will augment clinical knowledge and basic equipment requirements, but also aid clinicians, managers, and policy-makers to advocate for improvements in the quality of emergency care of children. This in turn will promote more formal development of PEM systems at local and national levels.
